# Regarding cornual pregnancy as a rare entity of ectopic pregnancy: A case report

**DOI:** 10.1016/j.ijscr.2024.109574

**Published:** 2024-03-27

**Authors:** Ainur Donayeva, Ibrahim A. Abdelazim, Ainur Amanzholkyzy

**Affiliations:** a Department of Normal Physiology, West Kazakhstan Marat Ospanov Medical University, Aktobe, Kazakhstan; b Department of Obstetrics and Gynecology, Faculty of Medicine Ain Shams University, Cairo, Egypt

**Keywords:** Cornual pregnancy, Ectopic, Interstitial pregnancy

## Abstract

The published Toumi et al’s article is somewhat confusing to the readers. The cornual pregnancy (CP) defined as a pregnancy that occurs in a rudimentary horn of a uterus with a Müllerian anomaly according to William's textbook. The interstitial ectopic pregnancy (IEP) occurs in the interstitial part of the fallopian tube where it crosses the uterine muscular to enter the uterine cavity.

The IEP sonographic findings include an empty uterus with an eccentrically placed gestational sac, located ≥1 cm from the endometrial margin and bordered by ≤ 5 mm myometrial rim.

Respectable Editor

Regarding the published article cornual pregnancy as a rare entity of ectopic pregnancy: A case report. doi: https://doi.org/10.1016/j.ijscr.2024.109364.

Toumi et al. [1] described in their case report, a case of cornual pregnancy (CP) with a sonographic finding of an empty uterus and an eccentrically placed gestational sac, bordered by a myometrial rim, separated by >1 cm from the empty uterine cavity, and contains a viable fetus with a crown-cranial length of 11 gestational weeks.

The published Toumi et al’s [1] article is somewhat confusing to the readers. The CP defined as a pregnancy that occurs in a rudimentary horn of a uterus with a Müllerian anomaly according to William's textbook [[2], [3], [4]].

Toumi et al’s [1] Fig. 1 is a sonographic image of interstitial ectopic pregnancy (IEP) which occurs in the interstitial part of the fallopian tube (FT) where it crosses the uterine muscular to enter the uterine cavity [5,6].

**Fig. 1:**
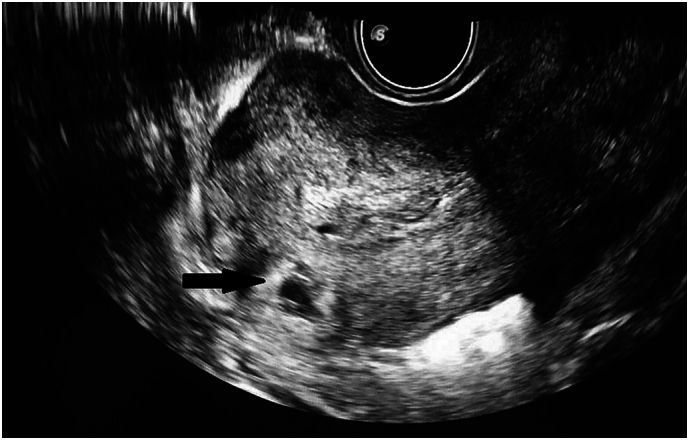
Toumi et al.'s figure 1 is a sonographic image of interstitial ectopic pregnancy (IEP).

The IEP sonographic findings include an empty uterus with an eccentrically placed gestational sac, located >1 cm from the endometrial margin and bordered by <5 mm myometrial rim [7]. The interstitial line (IL) is an echogenic line that extends from the gestational sac to the endometrial margin and represents the interstitial part of the FT (IL has 80 % sensitivity for diagnosing IEP) [8]. Additionally, Toumi et al.'s [1] Fig. 2 is an intraoperative image of IEP.

**Fig. 2:**
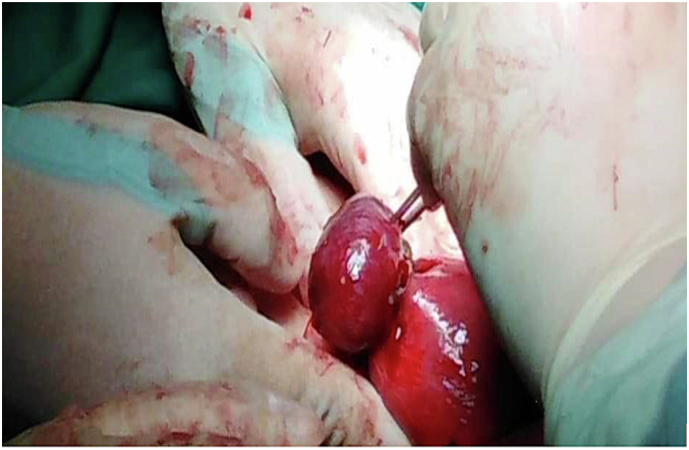
Toumi et al.'s figure 2 is an intraoperative image of interstitial ectopic pregnancy (IEP).

## Abbreviations

CP: Cornual pregnancy
FT: Fallopian tube
IEP: Interstitial ectopic pregnancy
IL: Interstitial line

## Ethics approval and consent to publication

Not applicable.

## Funding

Nil.

## Author contribution

AD, IAA, and AA designed this letter to editor, reviewed the literature, and revised this comment to editor before submission for publication.

## Guarantor

Ibrahim A. Abdelazim

## Research registration number

Not applicable.

## Conflict of interest statement

The authors declare no conflicts of interest.
